# Supplementary material for: Postural Balance Effects Associated with 400, 4000 or 10,000 IU Vitamin D3 Daily for Three Years: A Secondary Analysis of a Randomized Clinical Trial
Source: Nutrients. 2020 Feb 19;12(2):527. doi: 10.3390/nu12020527 (PMC7071365; doi:10.3390/nu12020527)
Supplement: Supplementary file 1 [file nutrients-12-00527-s001.pdf]

Supplementary Material

Figure S1. Flow diagram of participants in the study

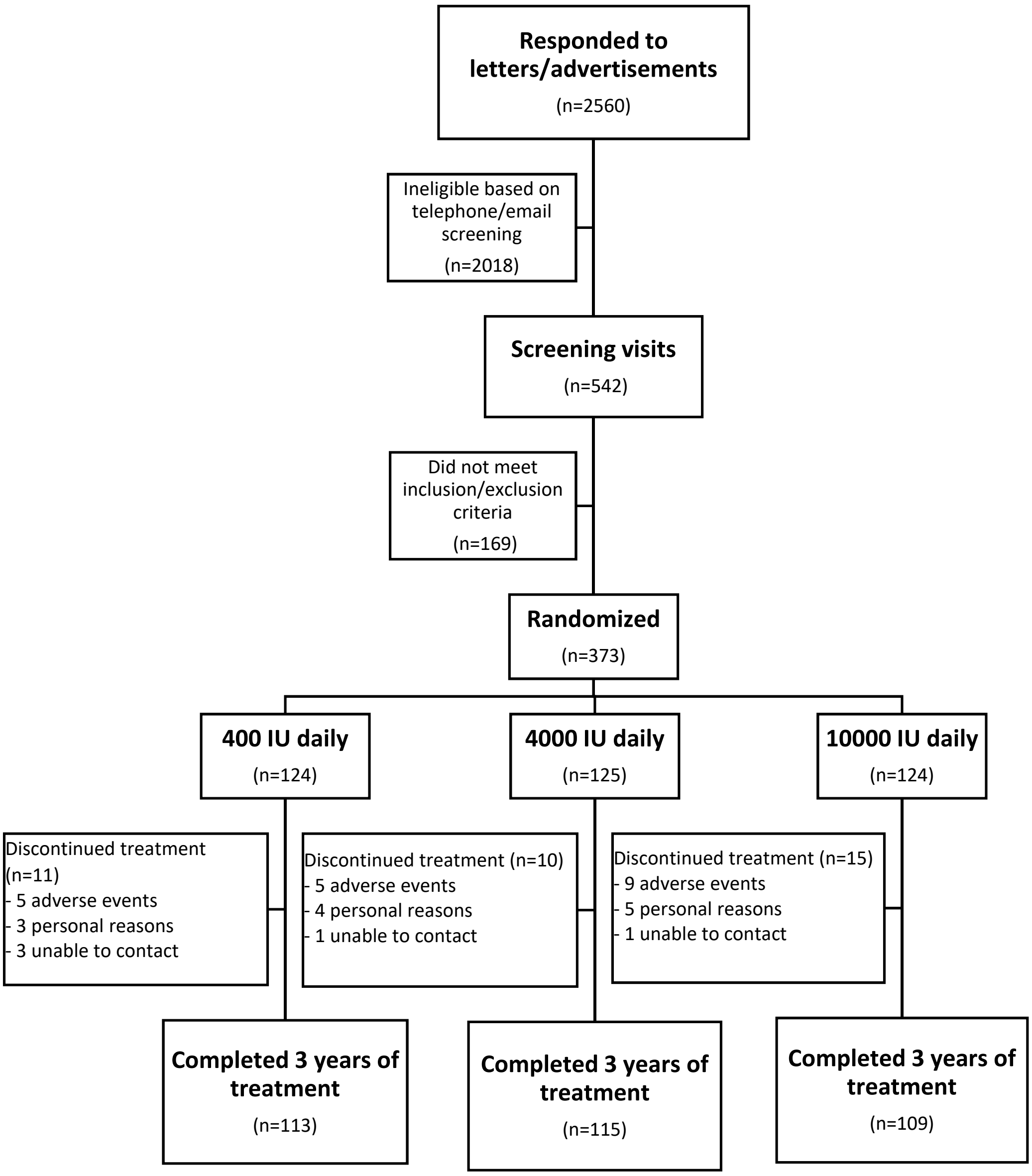

Supplement Table S1. Baseline and absolute mean change (SD) from baseline by treatment group for **medio-lateral (ML)** sway index.

		$SWAY_{EOFI}$	$SWAY_{ECFI}$	$SWAY_{EOFO}$	$SWAY_{ECFO}$
Baseline	400 IU	0.2 (0.1)	0.2 (0.1)	0.4 (0.1)	1.0 (0.3)
	4000 IU	0.1 (0.0)	0.2 (0.1)	0.4 (0.1)	1.0 (0.3)
	10000 IU	0.2 (0.1)	0.2 (0.1)	0.4 (0.1)	1.0 (0.3)
Δ 12-month	400 IU	0.00 (0.0)	0.00 (0.1)	-0.01 (0.1)	-0.05 (0.2)[a]
	4000 IU	0.00 (0.0)	0.00 (0.0)	-0.01 (0.1)	-0.05 (0.2)[a]
	10000 IU	0.00 (0.0)	0.00 (0.1)	-0.02 (0.1)	-0.09 (0.2)[a]
Δ 24-month	400 IU	0.00 (0.1)	0.00 (0.1)	-0.01 (0.1)	-0.08 (0.2)[a]
	4000 IU	0.01 (0.1)	0.00 (0.1)	0.00 (0.1)	-0.10 (0.2)[a]
	10000 IU	0.00 (0.1)	0.00 (0.1)	-0.01 (0.1)	-0.10 (0.2)[a]
Δ 36-month	400 IU	0.01 (0.1)	0.02 (0.1)	-0.01 (0.1)	-0.08 (0.3)[a]
	4000 IU	0.01 (0.1)	0.01 (0.1)	-0.01 (0.1)	-0.09 (0.3)[a]
	10000 IU	0.01 (0.1)	0.01 (0.1)	0.00 (0.1)	-0.13 (0.2)[a]

EOFI eyes open firm surface; ECFI eyes closed firm surface; EOFO eyes open foam surface; ECFO eyes closed foam surface. $p<0.05$ significantly different from [a]baseline.

Supplement Table S2. Baseline and absolute mean change (SD) from baseline by treatment group for **anterior-posterior (AP)** sway index.

		$SWAY_{EOFI}$	$SWAY_{ECFI}$	$SWAY_{EOFO}$	$SWAY_{ECFO}$
Baseline	400 IU	0.4 (0.1)	0.7 (0.2)	1.9 (0.4)	2.9 (2.0)
	4000 IU	0.4 (0.1)	0.7 (0.2)	2.0 (0.5)	3.1 (2.2)
	10000 IU	0.4 (0.1)	0.7 (0.2)	1.9 (0.5)	3.0 (2.2)
Δ 12-month	400 IU	0.00 (0.1)	0.00 (0.2)	-0.08 (0.3)	-0.18 (2.0)[ad]
	4000 IU	0.01 (0.1)	-0.01 (0.2)	-0.09 (0.3)	-0.20 (2.4)[ad]
	10000 IU	0.00 (0.1)	0.00 (0.2)	-0.08 (0.3)	-0.11 (2.1)[ad]
Δ 24-month	400 IU	-0.02 (0.1)	0.00 (0.2)	-0.12 (0.3)[ab]	-0.40 (1.8)[a]
	4000 IU	-0.01 (0.1)	0.01 (0.2)	-0.10 (0.3)[ab]	0.04 (2.1)[a]
	10000 IU	-0.01 (0.1)	0.00 (0.2)	-0.15 (0.4)[ab]	0.25 (2.1)[a]
Δ 36-month	400 IU	-0.01 (0.1)	0.00 (0.2)	-0.17 (0.3)[ab]	0.17 (2.2)[ab]
	4000 IU	0.00 (0.1)	0.00 (0.2)	-0.14 (0.4)[ab]	0.49 (2.1)[ab]
	10000 IU	0.00 (0.1)	0.01 (0.2)	-0.16 (0.4)[ab]	0.44 (2.1)[ab]

EOFI eyes open firm surface; ECFI eyes closed firm surface; EOFO eyes open foam surface; ECFO eyes closed foam surface. $p<0.05$ significantly different from [a]baseline, [b]12-months, [c]24-months, [d]36-months.

Supplement Table S3. Adjusted mean absolute difference (95% confidence interval) in **medio-lateral (ML)** sway index between treatment groups using linear mixed effects modeling.

		$SWAY_{EOFI}$	$SWAY_{ECFI}$	$SWAY_{EOFO}$	$SWAY_{ECFO}$
12-month difference	4000 – 400	-0.009 (-0.02, 0.01)	-0.010 (-0.04, 0.02)	0.019 (-0.02, 0.05)	0.038 (-0.04, 0.12)
	10000 – 400	-0.010 (-0.03, 0.01)	-0.004 (-0.03, 0.02)	-0.014 (-0.05, 0.02)	-0.018 (-0.10, 0.06)
	10000 – 4000	-0.001 (-0.02, 0.01)	0.006 (-0.02, 0.03)	-0.033 (-0.07, 0.00)	-0.056 (-0.14, 0.03)
24-month difference	4000 – 400	0.001 (-0.01, 0.02)	-0.005 (-0.03, 0.02)	0.028 (-0.01, 0.06)	0.015 (-0.07, 0.10)
	10000 – 400	0.001 (-0.01, 0.02)	-0.003 (-0.03, 0.02)	-0.007 (-0.04, 0.03)	-0.006 (-0.09, 0.08)
	10000 – 4000	0.000 (-0.02, 0.02)	0.001 (-0.03, 0.03)	-0.035 (-0.07, 0.00)	-0.021 (-0.10, 0.06)
36-month difference	4000 – 400	-0.005 (-0.02, 0.01)	-0.022 (-0.05, 0.01)	0.019 (-0.02, 0.05)	0.026 (-0.06, 0.11)
	10000 – 400	-0.002 (-0.02, 0.01)	-0.016 (-0.05, 0.01)	0.007 (-0.03, 0.04)	-0.025 (-0.11, 0.06)
	10000 – 4000	0.003 (-0.01, 0.02)	0.006 (-0.02, 0.03)	-0.013 (-0.05, 0.02)	-0.051 (-0.14, 0.03)

EOFI eyes open firm surface; ECFI eyes closed firm surface; EOFO eyes open foam surface; ECFO eyes closed foam surface. No significant effect of group or group by time interaction.

Supplement Table S4. Adjusted mean absolute difference (95% confidence interval) in **anterior-posterior (AP)** sway index between treatment groups using linear mixed effects modeling.

		$SWAY_{EOFI}$	$SWAY_{ECFI}$	$SWAY_{EOFO}$	$SWAY_{ECFO}$
12-month difference	4000 – 400	0.008 (-0.02, 0.04)	0.010 (-0.06, 0.08)	0.004 (-0.04, 0.05)	0.051 (-0.08, 0.18)
	10000 – 400	0.005 (-0.03, 0.04)	0.000 (-0.07, 0.07)	-0.001 (-0.04, 0.04)	0.018 (-0.11, 0.15)
	10000 – 4000	0.013 (-0.04, 0.03)	0.027 (-0.08, 0.06)	0.018 (-0.05, 0.04)	0.054 (-0.16, 0.10)
24-month difference	4000 – 400	0.009 (-0.02, 0.04)	0.026 (-0.04, 0.09)	0.011 (-0.03, 0.05)	0.073 (-0.05, 0.20)
	10000 – 400	0.005 (-0.03, 0.04)	-0.007 (-0.07, 0.06)	-0.023 (-0.07, 0.02)	-0.010 (-0.14, 0.11)
	10000 – 4000	0.013 (-0.04, 0.03)	0.028 (-0.10, 0.03)	0.018 (-0.08, 0.01)	0.052 (-0.21, 0.04)
36-month difference	4000 – 400	0.017 (-0.02, 0.05)	0.018 (-0.05, 0.08)	0.003 (-0.04, 0.05)	0.092 (-0.03, 0.21)
	10000 – 400	0.016 (-0.02, 0.05)	-0.001 (-0.07, 0.07)	-0.024 (-0.07, 0.02)	0.029 (-0.09, 0.15)
	10000 – 4000	0.014 (-0.03, 0.03)	0.028 (-0.08, 0.05)	0.018 (-0.07, 0.02)	0.050 (-0.18, 0.06)

EOFI eyes open firm surface; ECFI eyes closed firm surface; EOFO eyes open foam surface; ECFO eyes closed foam surface. No significant effect of group or group by time interaction.
